# Clinical manifestation of regional abnormal cortical plasticity: do dystonia and chronic pain have more in common than meets the eye?

**DOI:** 10.3389/fneur.2026.1843230

**Published:** 2026-06-10

**Authors:** Abteen Mostofi, Keyoumars Ashkan

**Affiliations:** 1 Department of Neurosurgery, Queen's Hospital, Romford, United Kingdom; 2 Department of Neurosurgery, King's College Hospital, London, United Kingdom

**Keywords:** cerebral cortex, chronic pain, dystonia, neuromodulation, neuroplasticity

## 1 Introduction

Neuroplasticity describes the nervous system's ability to reorganize itself in response to intrinsic or extrinsic stimuli and is critical for learning as well as recovery from injury. However, when this plasticity becomes excessive or maladaptive, it may be associated with the development of pathological conditions. Chronic pain and dystonia, though distinct disease entities, exemplify two such conditions in which maladaptive neuroplasticity, specifically in the sensorimotor areas of cerebral cortex, can be shown to exist.

Pain can be defined as an unpleasant sensory and emotional experience associated with actual or potential tissue damage. When persisting or recurring over more than 3 months it is known as chronic pain (1). Chronic pain etiology is diverse and can include pain of primarily nociceptive or neuropathic origin as well as “nociplastic” pain, a more recently recognized and poorly understood category of pain neither purely nociceptive nor neuropathic (2). Dystonia, however, is an umbrella term for movement disorders characterized by sustained or intermittent muscle contractions causing abnormal, often repetitive, movements, postures, or both. Dystonias can be further classified based on various clinical characteristics including distribution of affected body part(s) as well as etiology (3).

In this article, we briefly review the main threads of evidence exposing broad similarities between both conditions in the presence of maladaptive plasticity in the somatosensory and motor cortex. Broadly, these can be grouped into evidence demonstrating cortical reorganization, excessive synaptic plasticity and impaired inhibitory control. We also discuss the therapeutic implications of maladaptive plasticity particularly in relation to neuromodulatory therapies, particularly deep brain stimulation (DBS).

## 2 Cortical reorganization

Intrinsic to somatosensory and motor cortical areas is the existence of somatotopic maps. These maps are not fixed and can be modified by experience in a process very likely mediated by synaptic plasticity (4). Reorganization of somatotopic maps in somatosensory cortex is now well known to occur in a number of chronic pain states including chronic back pain, fibromyalgia, complex regional pain syndrome (CRPS), phantom limb pain and

carpal tunnel syndrome (5–9). This reorganization can take different forms depending on the etiology of pain. For example, in chronic back pain and fibromyalgia–types of nociceptive and nociplastic pain, respectively–the cortical representation of the back increases in size with chronicity (8). However, in neuropathic pain conditions CRPS type 1 and painful carpal tunnel syndrome, the opposite is seen, with reductions in the size of the cortical representation of the affected body part (7, 10). Furthermore, in phantom limb and other neuropathic pain syndromes, the extent of cortical reorganization is correlated with the intensity of pain experienced (8).

Similarly in focal dystonias (i.e., dystonia affecting a single body part), cortical representation abnormalities have been observed in both motor and primary somatosensory cortices in relation to the affected body part. For example, in patients with focal hand dystonia, transcranial magnetic stimulation (TMS) demonstrated enlarged motor cortical representations of the hand muscles compared to healthy controls (11), while functional neuroimaging revealed similar abnormalities in finger representation in the primary somatosensory cortex (12).

## 3 Excessive synaptic plasticity

Neurophysiological studies examining experimentally induced synaptic plasticity in patients also reveal parallels between chronic pain and dystonia. Common paradigms for inducing plasticity include paired associative stimulation (PAS) in which cortical stimulation using TMS is paired with a peripheral stimulus, as well as repetitive TMS (rTMS). In patients with focal hand dystonia, PAS using median nerve stimulation leads to increased plastic changes (potentiation or depression, depending on the relative timing of the stimuli) of motor-evoked potentials (MEPs) in the hand compared to healthy controls (13). Similarly, in patients with DYT1 dystonia (the most common genetic form of dystonia, usually generalized) and idiopathic cervical dystonia, rTMS-induced depression of MEPs was significantly more long lasting than in healthy controls (14).

It is broadly recognized that synaptic plasticity at several sites plays a major role in central sensitization which contributes to the development and maintenance of chronic pain (15). Evidence for this comes largely in the form of structural changes at cellular level and functional neuroimaging (16). *In vivo* neurophysiological examination of sensorimotor cortical plasticity in patients with chronic pain are few. A recent study in a cohort of patients with chronic pain, including those with neuropathic and mixed pain subtypes, utilized noxious laser heat in a PAS paradigm (17). While healthy controls showed long-lasting potentiation of TMS-induced MEPs in response to PAS, the chronic pain group exhibited abnormal MEP depression, suggesting abnormalities of plasticity in the motor cortex.

## 4 Deficient inhibitory control

Inhibitory neurotransmission within the cerebral cortex is a key determinant of neuroplasticity and learning. A decrease in local inhibitory signaling mediated by the main inhibitory neurotransmitter GABA is thought to enable plasticity to occur (18). Inhibition within the human motor cortex can be studied with TMS using short-interval intracortical inhibition (SICI) paradigms in which the delivery of a weak conditioning stimulus 1–5 ms prior to a test stimulus reduces the amplitude of the MEP evoked by the latter. SICI is thought to be related to GABA-mediated inhibition (19).

In both patients with chronic pain and focal hand dystonia, SICI has been demonstrated to be impaired, pointing to underlying disinhibition within the motor cortex (20–22). Within chronic pain, the effect seems more pronounced in neuropathic pain subtypes (22, 23). This disinhibited state may predispose to excessive or maladaptive neuroplasticity.

## 5 Discussion

### 5.1 Limitations of the current evidence

Chronic pain and dystonia are both conditions heterogeneous in etiology and phenotype. Markers of aberrant sensorimotor cortical plasticity feature in pain and dystonia of different etiologies and subtypes, though not uniformly. For example, cortical disinhibition appears more specific to neuropathic rather than nociceptive pain (23). Furthermore, changes in cortical reorganization are largely demonstrated in the context of focal dystonias and focal pain syndromes (though differently in neuropathic vs. nociceptive/nociplastic pain) where this is experimentally tractable based on the body parts affected. There are therefore differences between pain and dystonia subtypes in the way aberrant cortical plasticity manifests and how these relate to the pathophysiology is not currently clear.

There are some limitations in conclusions drawn from studies involving TMS, which form a large part of the evidence. First, responses to TMS protocols are known to be highly variable (24). Sources of inter- and intra-individual variability are common and include age, genetics, sleep and blood glucose amongst others (25). TMS-based studies typically involve small numbers of patients and healthy controls and factors giving rise to variability are unlikely to be well controlled. There are therefore implications of this variability on the utility of TMS as a reliable diagnostic tool (26). Especially within the dystonia field, the literature on TMS studies of abnormal plasticity remain the source of ongoing debate. In addition to the studies described above which demonstrate differences in cortical plasticity in dystonia patients vs. healthy controls, there are others which have not reproduced this finding (24). Furthermore, recorded changes in cortical excitability in TMS protocols are only indirect and inferred measures of plasticity occurring at the level of the synapse.

Dystonia and chronic pain are both recognized to be network disorders (27, 28). While we have focused at level of the sensorimotor cortex, it is likely that cortical changes are not isolated and interact with neuroplasticity occurring at other sites in the network (29). Understanding the role of sensorimotor cortical plasticity within the wider network, however, is a crucial part of elucidating its mechanism and pathophysiological relevance.

Finally, what has not been demonstrated is the causal relationship of maldaptive cortical neuroplasticity to the phenotype of chronic pain and dystonia. The aberrant changes observed may instead represent an effect of the phenotype, some form of compensatory mechanism, or an epiphenomenon with no pathophysiological role.

### 5.2 Therapeutic implications

Given the shared features of maladaptive plasticity in chronic pain and dystonia, it is an attractive hypothesis that these changes play some causal role and that the modulation or “resetting” of aberrant plasticity might be a promising therapeutic approach for both conditions. Deep brain stimulation (DBS) is now an established therapy for medically refractory dystonia (30) and has been used to good effect in many refractory chronic pain conditions (31). The evidence base for DBS in primary (idiopathic and genetic) dystonias is strong and is supported by randomized controlled trials (32), whereas in chronic pain it is more variable and based around case series (32, 33). This may reflect heterogeneity of chronic pain syndromes in the patients included in these studies. Even within the dystonias, efficacy of DBS can be variable depending on etiology with secondary dystonias known to respond less predictably (34).

In dystonia, DBS of the globus pallidus pars interna (GPi; the main evidence-based brain target for dystonia) is well known to exhibit a delay of weeks or months before clinical benefit is seen, unlike DBS for other movement disorders such as Parkinson's disease or essential tremor, for example, where the therapeutic effects on motor symptoms have almost instantaneous onset. This finding may be interpreted as signifying a therapeutic role for DBS in resetting the aberrant plasticity in the responsible cortico-subcortical brain networks (35). In further evidence of DBS modulating plasticity in dystonia, GPi DBS in patients with primary generalized dystonia prevented the potentiation of MEPs seen when patients underwent PAS using a median nerve stimulus (36). This effect correlated with the clinical response to DBS and suggests a therapeutic role for the effects of DBS on motor cortex plasticity. In contrast, the relationship of DBS-induced cortical plasticity and therapeutic benefit in chronic pain has not been well studied.

Though not part of routine clinical practice, the use of non-invasive neuromodulation techniques such as transcranial magnetic or electrical stimulation may have some benefit in dystonia and chronic pain, though effects are variable and the duration of effect does not appear to be long-lasting (37, 38). Any benefit from these approaches is thought to be related to modulation of neuroplasticity and it has been suggested that focusing on the underlying mechanistic aspects of this can help improve the efficacy of this group of therapies in the future (15, 39).

### 5.3 Convergence of treatment strategies and future directions

Chronic pain and dystonia appear to share pathophysiological characteristics of altered or maladaptive neuroplasticity in the sensorimotor cortex. The recognition of these common features opens avenues for cross-disciplinary treatment approaches. In particular, DBS shows promise in dystonia by exerting its therapeutic effect in association with alteration of motor cortical plasticity. The impact of DBS on sensorimotor cortical plasticity in chronic pain has not been studied though would seem a promising avenue to investigate in order to optimize therapeutic benefits in a treatment where the non-responder rate can be high depending on the specific etiology (40).

Interestingly, chronic pain is known to be very prevalent in dystonia. Evidence is emerging of underlying central sensory processing abnormalities which potentially contribute to pain in primary dystonia (41, 42). In addition to improving motor symptoms, DBS for dystonia is known to improve pain (43, 44), though how much of this analgesic effect is related to the impact on cortical neuroplasticity has not been well studied.

Future research should be directed at elucidating the role of dysregulated cortical neuroplasticity in chronic pain and dystonia. If a causal relationship could be demonstrated, then this might provide a novel therapeutic target for neuromodulation. Greater knowledge of neuroplastic mechanisms and their modulation by techniques such as DBS, could help refine patient selection criteria and optimize stimulation parameters specifically to address maladaptive plasticity. Even in the absence of a clear causal role, it is possible that there may still be utility in using measures of aberrant plasticity as biomarkers of therapeutic efficacy if the relationship with the phenotype proves to be robust. Such approaches could be used in synergy with other therapies–for example, pharmacological, physical, and psychological–to provide multifaceted, individualized approaches to target maladaptive neuroplasticity in order to maximize therapeutic efficacy.
